# Multispacer Typing of *Bartonella henselae* Isolates from Humans and Cats, Japan

**DOI:** 10.3201/eid1612.100962

**Published:** 2010-12

**Authors:** Masashi Yanagihara, Hidehiro Tsuneoka, Motoki Sugasaki, Junzo Nojima, Kiyoshi Ichihara

**Affiliations:** Author affiliation: Yamaguchi University, Ube, Yamaguchi, Japan

**Keywords:** Bacteria, zoonoses, Bartonella henselae, bartonellosis, cat-scratch disease, bacterial typing, intergenic sequence, multispacer typing, Japan, dispatch

## Abstract

To determine genotypic distribution of and relationship between human and cat strains of *Bartonella henselae,* we characterized 56 specimens using multispacer typing (MST). Of 13 MST genotypes identified, 12 were grouped into cluster 1. In Japan, human infections can be caused by *B. henselae* strains in cluster 1.

The causative agent of cat-scratch disease (CSD), *Bartonella henselae*, is a gram-negative bacterium associated with cats. Human infection usually occurs through scratches or bites by infected cats and typically is seen with localized lymphadenopathy. Occasionally, the infection may have an atypical manifestation, such as endocarditis, encephalopathy, neuroretinitis, or systemic CSD with hepatic and splenic granuloma (*1*).

*B. henselae* strains are classified into two 16S rRNA genotypes, 16S type I/Houston-1 and 16S type II/Marseille. Although both genotypes are present worldwide, 16S type II appears to be dominant in the cat population of Europe, whereas 16S type I is more common in Asia, including Japan (*2**,**3*).

Multispacer typing (MST) is a nucleotide sequencing-based genotyping method that uses highly variable intergenic spacers as typing markers. It is the most suitable genotyping procedure for evaluating the population structure of closely related strains of *B. henselae* (*4*). Previously, 50 MST genotypes from 201 *B. henselae* strains were phylogenetically organized into 4 lineages, and human strains mostly grouped within 2 of these lineages, Houston-1 and Marseille (*5**,**6*). Because genotypic data on *B. henselae* from Asian countries are limited, we applied MST to 56 human and cat specimens to determine the genotypic distribution and relationship of human and cat strains of *B. henselae* in Japan.

## The Study

During 1997 through 2008, we collected 56 *B. henselae* specimens from western Japan, mainly from Yamaguchi prefecture; the specimens included 1 *B. henselae* isolate from a patient with endocarditis (*7*), 24 clinical specimens from CSD patients who had test results positive for *B. henselae* DNA, and 31 *B. henselae* isolates from domestic cats (*8*). The 24 clinical specimens included 5 lymph node specimens and 16 pus specimens from patients with typical CSD, 1 blood specimen from a patient with bacteremia, 1 liver specimen from a patient with hepatic granuloma, and 1 spleen specimen from a patient with splenic granuloma. Total genomic DNA was extracted from the specimens by using the QIAamp DNA Mini Kit (QIAGEN, Hilden, Germany). *B. henselae* DNA was detected by using PCR with specific primers for the 16S–23S rRNA intergenic spacer (*9*) and the *htrA* gene (*10*), and the 16S rRNA genotype was confirmed by partial sequencing of the 16S rRNA gene (*11*).

In addition, previously described MST primers were used to amplify and sequence the 9 intergenic spacers in *B. henselae* DNA (*6*). Locus-specific PCR was performed for spacer S1; direct sequencing was unsuccessful because of an unusual number of variable number tandem repeats (VNTR) (*8*). Spacer sequences were assigned according to published data (*5**,**6**,**8*). For each of the 9 spacers (S1–S9), we identified 8, 2, 3, 4, 3, 2, 3, 2, and 3 genotypes, respectively (Table). Three novel spacer sequences were deposited in the DNA Data Bank of Japan with these accession numbers: AB558532, S2 genotype 9; AB558533, S4 genotype 7; and AB558534, S4 genotype 8. We identified 13 different MST genotypes among the 56 specimens (Table). Of these 13 MST genotypes, 7 were novel (types 51–57). Six MST genotypes belonged to human and cat strains, including 2 predominant MST genotypes (14 and 35). All MST data were deposited in the MST-Rick database (http://ifr48.timone.univ-mrs.fr/MST_BHenselae/mst).

**Table:** Multispacer typing of 56 *Bartonella henselae* strains isolated from humans and cats, Japan*

*B. henselae* source			Genotypes										16S rRNA genotype
No. human	No. cat	S1	S2	S3	S4	S5	S6	S7	S8	S9	MST
1	1		7	2	5	4	1	2	1	1	3	7	I
8	12		4	2	5	4	1	2	1	1	3	14	I
1	0		3	2	6	5	2	2	2	1	1	21	I
1	0		8	2	5	4	1	2	2	1	3	32	I
2†	1		4	2	5	4	1	2	2	1	3	33	I
9‡§	4		5	2	6	5	2	2	2	1	1	35	I
0	1		11	2	6	4	1	2	1	1	3	51	I
0	2		12	9	2	7	5	4	4	3	2	52	II
1	2		5	2	6	8	2	2	2	1	1	53	I
1¶	1		4	2	5	5	1	2	1	1	3	54	I
0	4		5	2	6	5	1	2	2	1	1	55	I
0	3		7 + 4#	2	5	4	1	2	1	1	3	56	I
1	0		4	2	5	4	2	2	1	1	3	57	I

*MST, multispacer typing; I, 16S type I; II, 16S type II. †Includes a clinical specimen isolated from a patient with hepatic granuloma. ‡Includes *B. henselae* strain isolated from a patient with endocarditis. §Includes a clinical specimen isolated from a patient with bacteremia. ¶Includes a clinical specimen isolated from a patient with splenic granuloma. #Strain with 2 different copies of intergenic spacer S1 in its genome.

Subsequently, we analyzed the phylogenetic relationships of the 7 novel MST genotypes identified in this study with the 50 previously identified genotypes. Multiple sequence alignment of the concatenated spacer sequences was performed by using ClustalW (www.ebi.ac.uk/clustalw). Finally, a phylogenetic tree was constructed by using the unweighted pair-group method with arithmetic mean (UPGMA) in MEGA4 (*12*). This phylogenetic tree is grouped into 4 clusters (Figure). Of the 13 MST genotypes identified in this study, 12 genotypes belonged to cluster 1, but one genotype (MST genotype 52) belonged to cluster 4.

**Figure:**
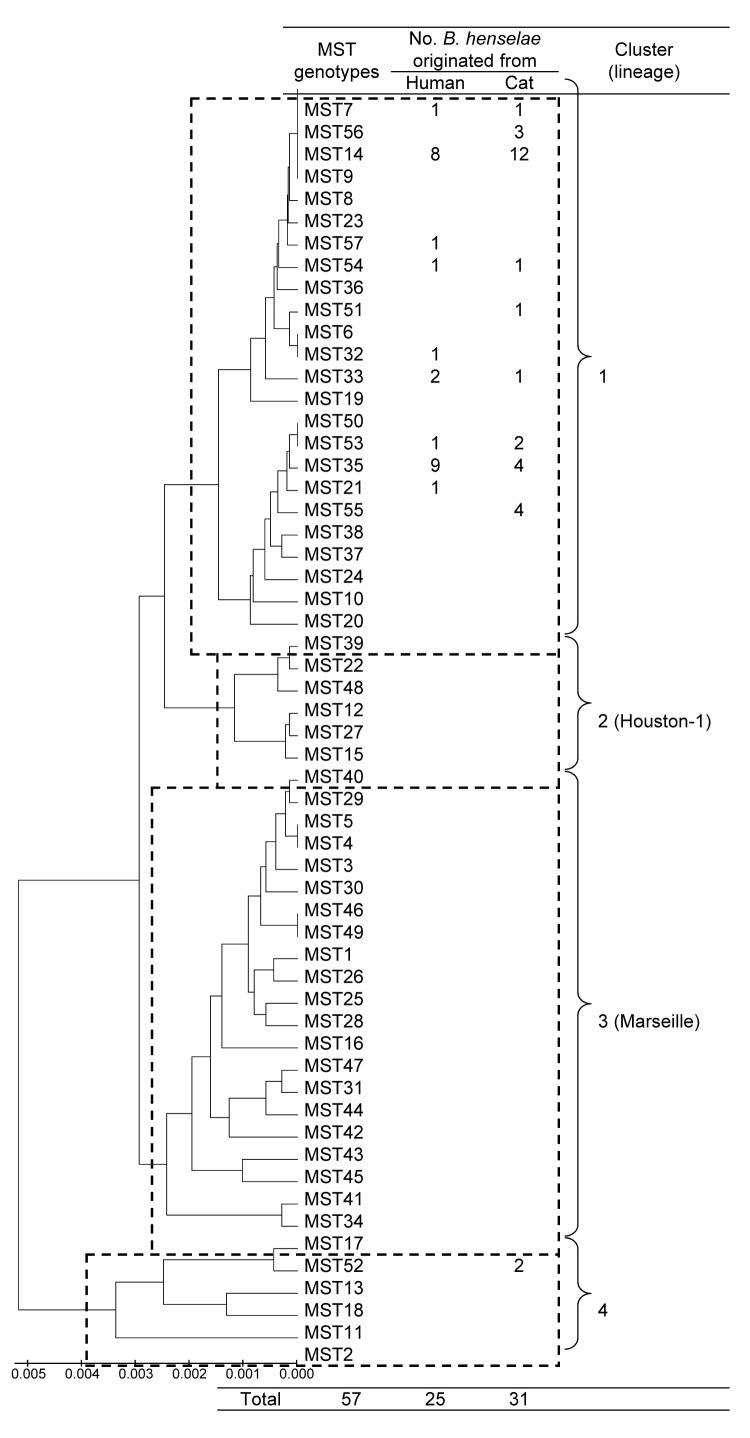
Phylogeny and clusters of multispacer typing (MST) genotypes of *Bartonella henselae* isolates from humans and cats, Japan, based on 9 concatenated intergenic spacer sequences in 57 MST genotypes. The unweighted pair-group method with arithmetic mean method in MEGA4 software (*12*) was used for phylogenetic analysis. Dotted rectangles show 4 clusters of MST genotypes, 2 of which correspond to the *B. henselae* Houston-1 and Marseille type strains. Scale bar indicates nucleotide substitutions per site.

## Conclusions

This study showed that MST genotypes in Japan were mainly grouped into 1 lineage (cluster 1), which was composed of Asian and American strains of *B. henselae*, and that the genotypic distribution of human strains coincided with that of cat strains. Although only 1 human strain from the West Indies belonged to cluster 1 before this study (*6*), we discovered that all 25 of our human strains from Japan were grouped into cluster 1. These results demonstrate that human infections can be caused by *B. henselae* strains in cluster 1, which differed from clusters corresponding to the Houston-1 and Marseille type strains.

In a previous study, MST genotype 35 was the most common genotype in cluster 1, and 4/6 (67%) of Japanese cat strains belonged to this genotype (*5*). In this study, MST genotypes 14 (36%; 20/56) and 35 (23%; 13/56) were predominant genotypes. Additionally, most human strains (88%; 15/17) belonging to these genotypes were isolated from patients with typical CSD; 2 strains with MST genotype 35 were isolated from patients with endocarditis and bacteremia (Table).

The genotypic distribution of the human strains in this study differed from that reported by Li et al. (*6*) because their strains isolated in France were grouped under 2 lineages (Houston-1 and Marseille). However, we found that the lineages of human strains matched those of cat strains in each country. These results are consistent with the role of cats as the major reservoir of *B. henselae* (*13*).

In this study, we identified 2 cat strains that were classified into cluster 4. These strains belonged to 16S type II, which is rare in Japan (*3*). In previous MST studies, strains in cluster 4 were isolated from cats and belonged to 16S type II (*5**,**6*). Intriguingly, similar lineages consisting of 16S type II isolates from cats were observed in other genotyping studies involving the use of multilocus sequence typing (MLST) (*14*) and multiple locus variable number tandem repeat analysis (MLVA) (*15*). Thus, these lineages may be less pathogenic for humans. However, further studies are needed to investigate this hypothesis.

When we characterized the strains in this study by MLST we found that almost all of them shared the same sequence type as Houston-1 (*8*). In contrast, we identified 13 MST genotypes that belonged to different clusters than Houston-1. The lower resolving power of MLST is mostly likely due to sequence conservation in the 8 housekeeping genes selected for the method. MST has a higher resolving power because the spacers used in this method are more variable than MLST markers. As a result, MST is better suited for evaluating the population structure of closely related *B. henselae* strains.

We conclude that the MST genotypes in Japan are mainly grouped into cluster 1 and that the genotypic distribution of human strains coincides with that of cat strains. In Japan, human infections can be caused by *B. henselae* strains in cluster 1, distinct from clusters containing the Houston-1 and Marseille type strains. These results improve our understanding of the population structure of and geographic relationship between human and cat strains of *B. henselae*.
